# Supplementary material for: A family of unconventional deubiquitinases with modular chain specificity determinants
Source: Nat Commun. 2018 Feb 23;9:799. doi: 10.1038/s41467-018-03148-5 (PMC5824887; doi:10.1038/s41467-018-03148-5)
Supplement: Supplementary file 1 — Supplementary Information [file 41467_2018_3148_MOESM1_ESM.pdf]

A family of unconventional deubiquitinases with modular chain specificity determinants

Hermanns et al.

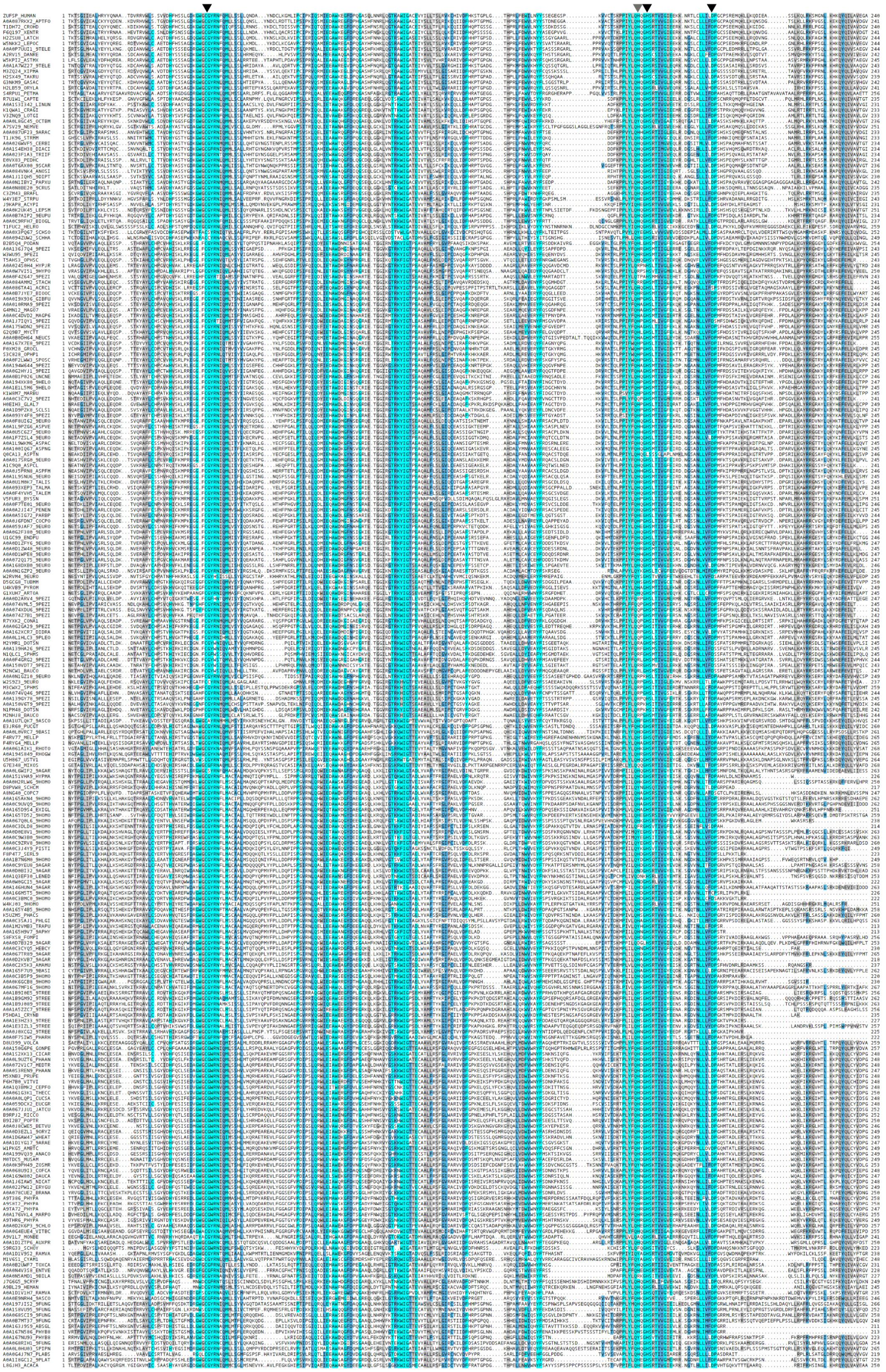

Supplementary Figure 1: Conservation of the ZUFSP/Mug105 family. Multiple alignment of the catalytic domain of representative ZUFSP family members from all eukaryotic kingdoms. Sequences are denoted by their Uniprot Accession numbers. Invariant and highly conserved positions are indicated by colors of different intensities. The active site residues are indicated by black triangles above the alignment. A second conserved His residue, which could later be excluded as active site residue is indicated by a grey triangle.

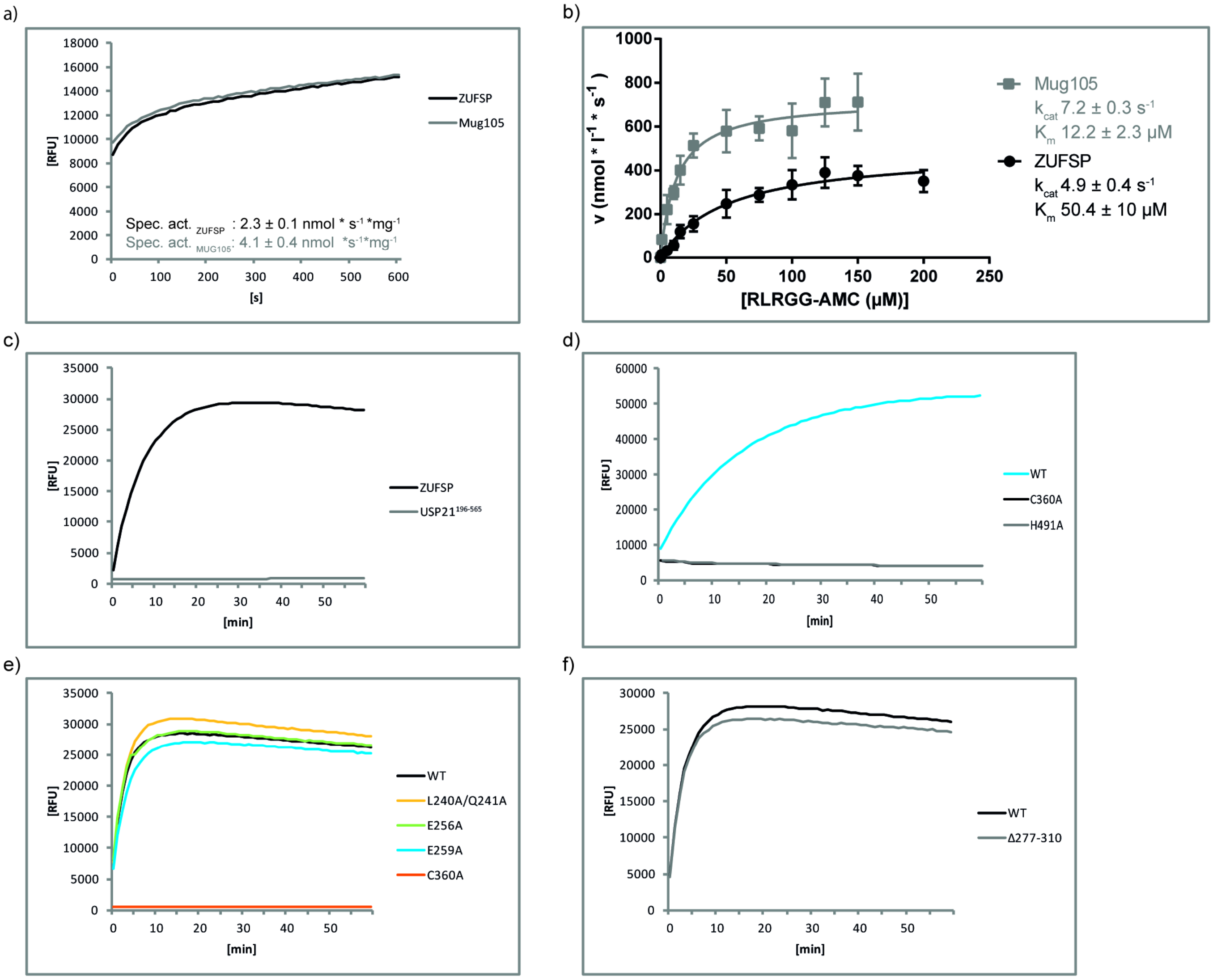

Supplementary Figure 2: Activity assays with fluorogenic substrates

a) Specific activity of ZUFSP and Mug105 against Ub-AMC was determined by using 100 nM DUB and 5 µM Ub-AMC. Initial rate was derived from the displayed curves and used to calculate the specific activity. The displayed RFU values are the means of triplicates.

b) Steady state kinetics using RLRGG-AMC as substrate at varying concentrations. Initial rates were plotted against the substrate concentration and a Michaels-Menten curve was fitted to the experimental data. Error bars represent the standard deviation of three independent measurements, each of them using 100 nM DUB.

c) Fluorometric activity assay of ZUFSP and USP21$^{196-565}$ using RLRGG-AMC as substrate. Released fluorescence (RFU) is measured over time. All RFU values are means of triplicates.

d) Fluorometric activity assay comparing active ZUFSP$^{232-578}$, inactive ZUFSP$^{232-578}$ C360A, and a mutant of the proposed catalytic histidine ZUFSP$^{232-578}$ H491A.

e) Fluorometric activity assay comparing ZUFSP$^{148-578}$, ZUFSP$^{148-578}$ C360A, and the mutant versions L240A/Q241A, E256A and E259A.

f) Fluorometric activity assay comparing ZUFSP$^{148-578}$ with ZUFSP$^{148-578, \Delta 277-310}$.

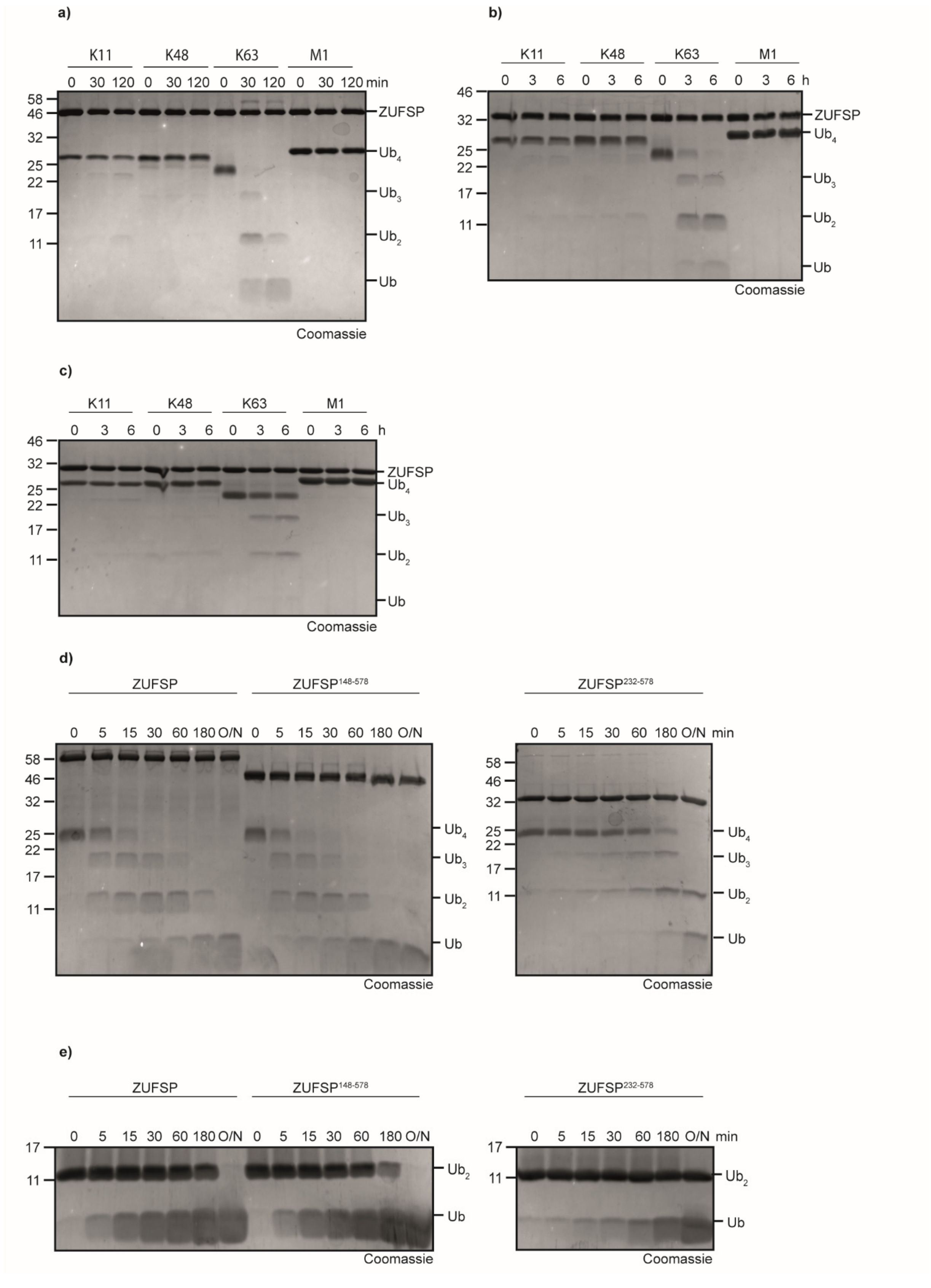

Supplementary Figure 3: UBDs of ZUFSP are important for activity but not for specificity.

Linkage specificity analysis with ZUFSP truncations. A panel of ZUFSP truncations a) ZUFSP$^{148\text{-}578}$, b) ZUFSP$^{232\text{-}578}$ and c) ZUFSP$^{249\text{-}578}$ were tested against a panel of differently linked tetra-ubiquitin species for the indicated times. Time course analysis of d) K63-linked Ub_4 chains and e) K63-linked Ub_2 chains cleaved by full length ZUFSP, ZUFSP$^{148\text{-}578}$ and ZUFSP$^{232\text{-}578}$.

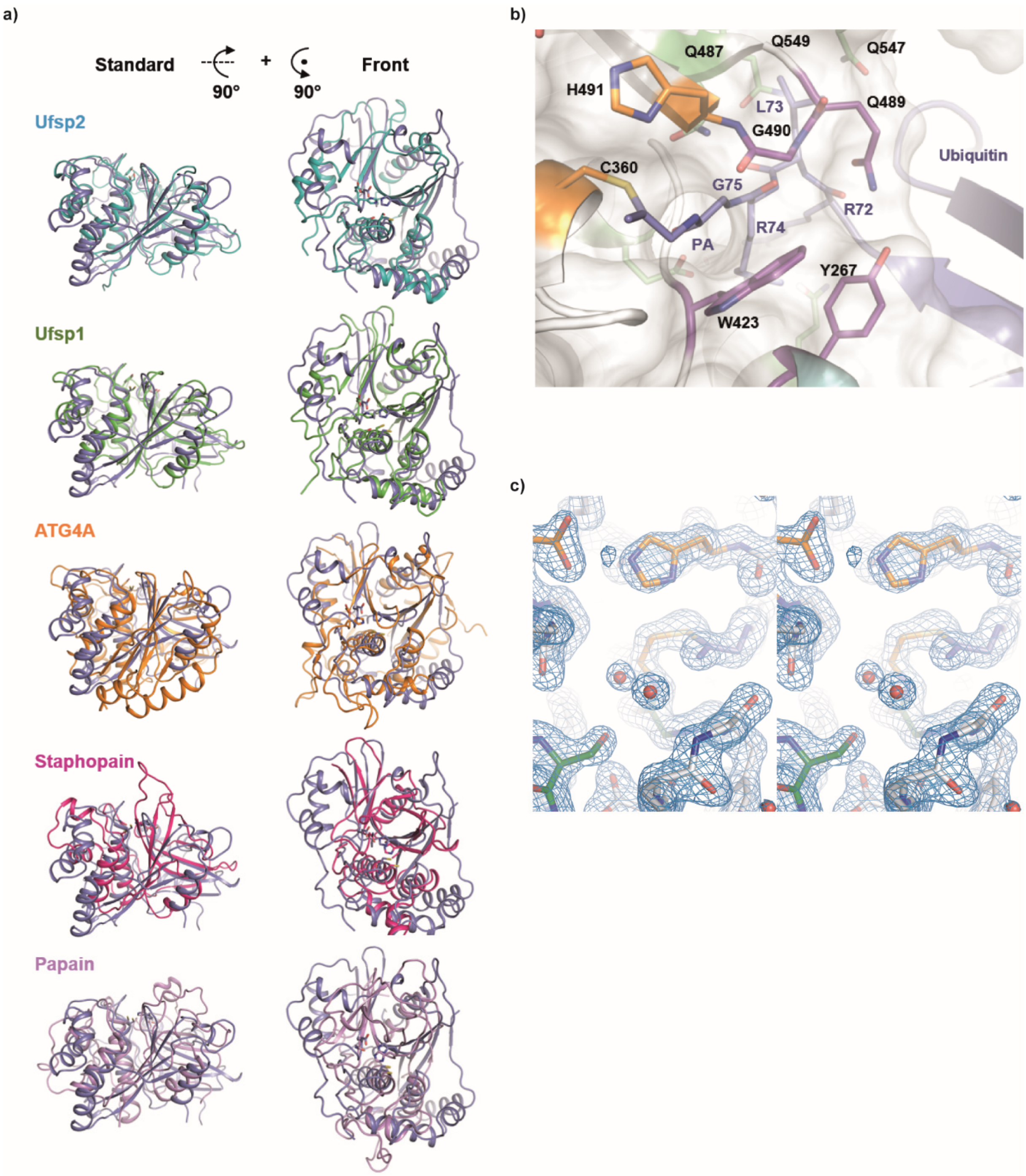

Supplementary Figure 4: Structural similarity of ZUFSP with related protease families and additional close-ups of key sites of ZUFSP

a) Structural comparison of the catalytic domain of ZUFSP with various related cysteine peptidases in two perspectives. Structures are shown in cartoon representation with the catalytic triad shown as sticks. The structures were superimposed using the CE algorithm of PyMOL. ZUFSP – Ufsp2 (over 200 residues with a R.M.S distance (RMSD) of 3.65 Å, ZUFSP – Ufsp1 (200 residues / RMSD: 3.98 Å), ZUFSP – ATG4A (168 residues / RMSD: 5.76 Å), ZUFSP – Staphopain (144 residues/ RMSD: 4.83 Å), ZUFSP – Papain (112 residues / RMSD: 4.4 Å). PDB IDs: 3OQC, 2Z84, 2P82, 1CV8, 6PAD

b) Magnification of the substrate binding groove at the unprimed site. Ubiquitin is colored blue and residues of the catalytic triad are in orange. ZUFSP residues required for recognition of ubiquitin at Arg-72, Leu-73 and Arg-73 recognition are colored green. Tyr-267, Trp-423, Gln-489 and Gly490 are colored purple and are forming a narrow tunnel around the C-terminal propargyl moiety (PA) and Gly-75 of ubiquitin.

c) Stereo view on the active site. The catalytic triad is colored orange; PA is in blue; putative components of the oxyanion hole in green. Remaining residues are in light grey. The experimental electron density map (2mFo-DFc) is shown as blue mesh at a contour level of 1.5σ (0.44 e/Å^3).

a)

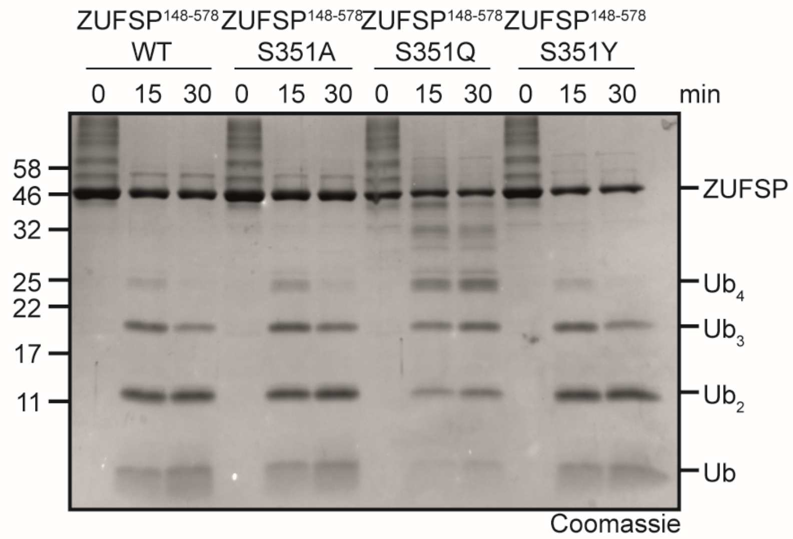

b)

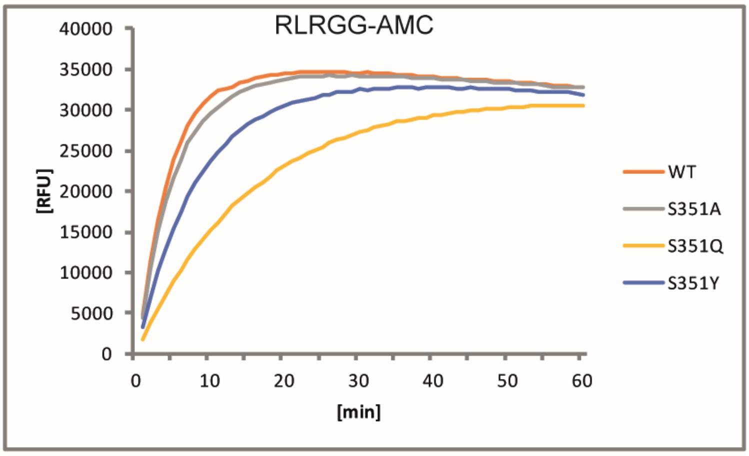

Supplementary Figure 5: Ser-351 is not involved in oxyanion hole formation

a) Activity of S351 mutants (ZUFSP$^{148\text{-}578}$ S351A, S351Q or S351Y) on K63-linked Ub_{6+} chains, in comparison to wildtype ZUFSP$^{148\text{-}578}$.

b) Activity of S351 mutants (ZUFSP$^{148\text{-}578}$ S351A, S351Q or S351Y) on RLRGG-AMC, in comparison to wildtype ZUFSP$^{148\text{-}578}$. The displayed RFU values are the means of triplicates.

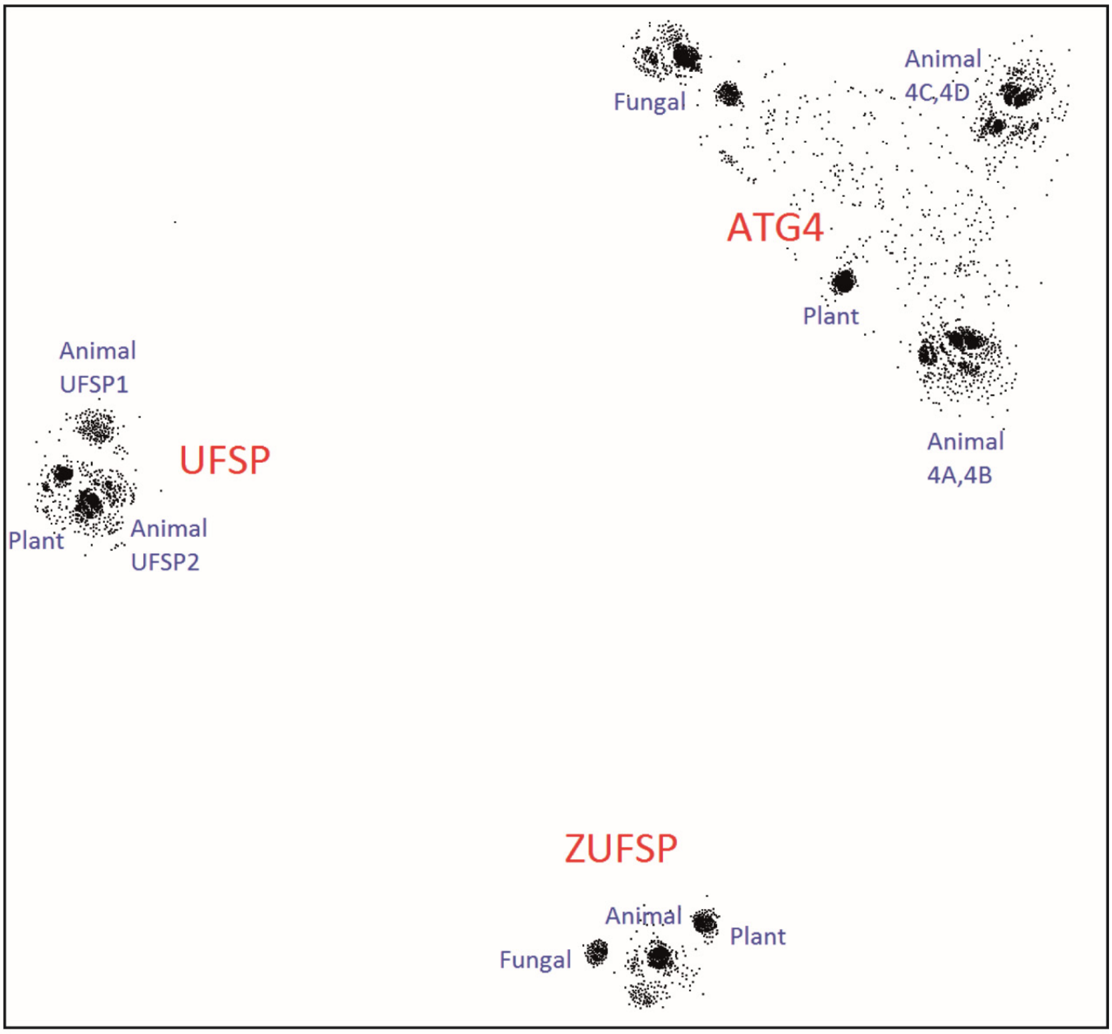

Supplementary Figure 6: Clustering analysis of ZUFSP, UFSP and ATG4 family members

All sequences with significant sequence similarity to the ZUFSP family (see suppl. Figure 1), together with members of the UFSP and ATG4 families, were subjected to clustering analysis with the CLANS program [1]. Each dot represents one sequence. Distance between the dots is scaled by sequence similarity, resulting in clusters of related sequences. The main families ZUFSP, UFSP and ATG4 are clearly distinct, certain subfamilies are also visible and are labeled.

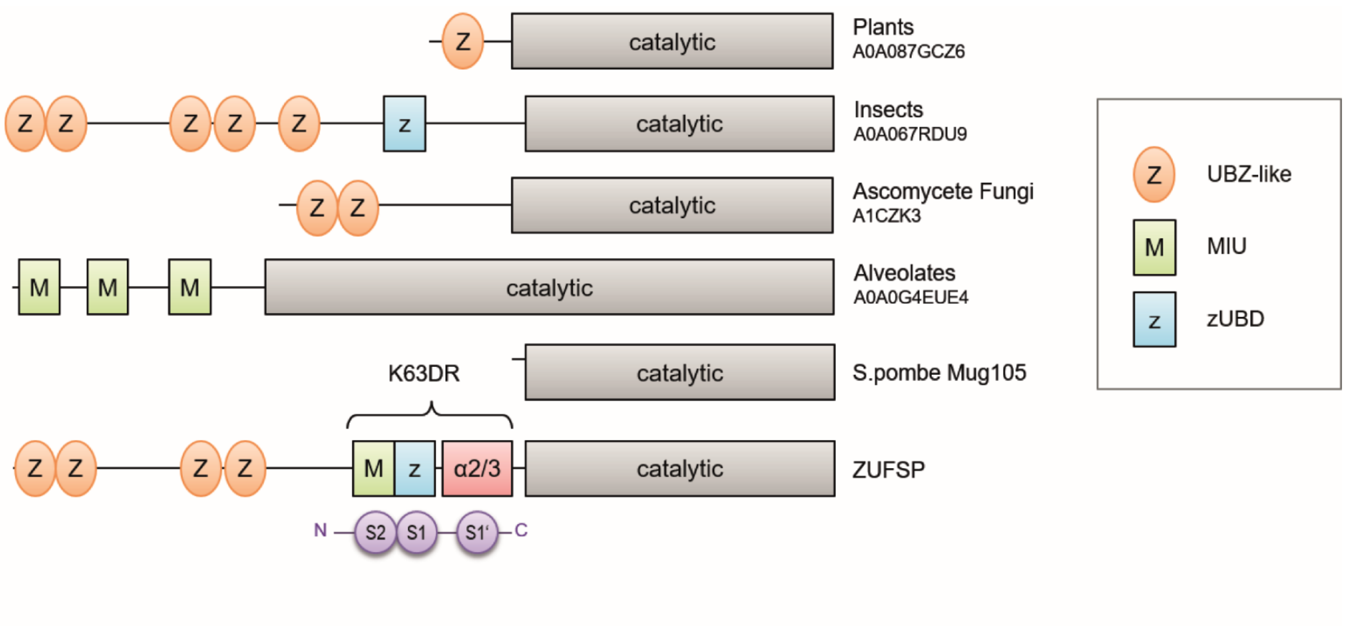

Supplementary Figure 7: Domain architecture of representative ZUFSP family members

The domain architecture of six representative ZUFSP family members is shown. UBZ-like Zn-fingers are shown as orange circles, MIU domains as green boxes, zUBD domains as blue boxes. The α2/3 helix region is specific to mammalian members and is shown as a red box. For human ZUFSP, the regions responsible for binding the distal (outgoing) ubiquitin units S1 and S2, as well as the proximal (substrate) ubiquitin unit S1’ are indicated.

primer name	sequence in 5'- 3'
pOPINS ZUFSP 1 fwd	GCGAACAGATCGGTGGTATGCTTTCCTGTAATATTTGTGGTGAAACAGTAACCTCAGAACC
pOPINS ZUFSP 148 fwd	GCGAACAGATCGGTGGTGAAACAACATACAGTCCTCCTGAATGTCCATTCTGTGG
pOPINS ZUFSP 232 fwd	GCGAACAGATCGGTGGTGGTGATCTACAATTGGCTCACCAGCTTCAGCAAG
pOPINS ZUFSP 249 fwd	GCGAACAGATCGGTGGTAGATCTGAAGAATCAAGACAAGAAATAGAAGAATTTCAGAAGCTGCAG
pOPINS ZUFSP 274 fwd	GCGAACAGATCGGTGGTGGATACAAACAACAACAACTACGAAATATGGAGATAGAAGTAAATAGGG
pOPINS ZUFSP 310 fwd	GCGAACAGATCGGTGGTCTTGGTTTTGACGATGGAAAAACAAAAACTTCCGGAATTATTGAAGC
pOPINS ZUFSP 578 fwd	ATGGTCTAGAAAGCTTTAAGGAATCTTCTCGGCTGTAAAGACTTGAGAAGCTTGTC
pCDNA5 FRT TO 3xFLAG-ZUFSP 1 fwd	TACCGAGCTCGGATCATGGACTACAAAGACCATGACGGTGATTATAAAGATCATGACATCGACTACAAGGATGACGATGACAAGATGCTTTCCTGTAATATTTGTGGTGAAACAGTAACCTCAGAACC
pCDNA5 FRT TO ZUFSP 578 rev	TAGACTCGAGCGGCCTTAAGGAATCTTCTCGGCTGTAAAGACTTGAGAAGCTTGTC
pOPINS Mug105 1 fwd	GCGAACAGATCGGTGGTATGTCAAAATGCTTGCAGCAGCTTAAACGACAACTTCAGC
pOPINS Mug105 244 rev	ATGGTCTAGAAAGCTTTAAAAGTCCGAAATTCTGGTAGAACGAACTTCCAGTTTTGAACTG
pTXB1 Ubiquitin 1 fwd	GGGGGGCATATGCAGATCTTCGTGAAGACCCTG
pTXB1 Ubiquitin 75 rev	GGTGGTTGCTCTTCCGCAACCTCTGAGACGGAGGACCAG
pTXB1 LC3B 1 fwd	GGGGGGCATATGCCGTCGGAGAAGACCTTCAAG
pTXB1 LC3B 119 rev	GGTGGTTGCTCTTCCGCAGAACGTCTCCTGGGAGGCATAG
ZUFSP C360A fwd	GGAAATTTCTGTAACCAGCACCCCAACCTTTGTCGCCTAAA
ZUFSP C360A rev	TTTAGGCGACAAAGGTTGGGGTGCTGGTTACAGAAATTTCC
ZUFSP D406A fwd	GAGGCCCCCTGAGGAGCAAAACCTTCCTTCC
ZUFSP D406A rev	GGAAGGAAGGTTTTGCTCCTCAGGGGGCCTC
ZUFSP E428A fwd	GTCAGGAGTATATATACTGCACATGCTCCAATCCAGG
ZUFSP E428A rev	CCTGGATTGGAGCATGTGCAGTATATATACTCCTGAC
ZUFSP H491A fwd	CAATTCCAATAACAGTTCGACTGGCACCTTGATGCTGAAGATAGATAG
ZUFSP H491A rev	CTATCTATCTTCAGCATCAAGGTGCCAGTCGAACTGTTATTGGAATTG
ZUFSP E256A fwd	GCAGCTTCTGAAATTCTTCTATTGCTTGTCTTGATTCTTCAGATCTC
ZUFSP E256A rev	GAGATCTGAAGAATCAAGACAAGCAATAGAAGAATTTCAGAAGCTGC
ZUFSP E259A fwd	GAATCAAGACAAGAAATAGAAGCATTTCAGAAGCTGCAGAGACAA
ZUFSP E259A rev	TTGTCTCTGCAGCTTCTGAAATGCTTCTATTTCTTGTCTTGATTC
ZUFSP L240A, Q241A fwd	TCCTCTTTCTGTCTTCTTCTTGCGCAGCCTGGTGAGCCAATTGTAGATCAC
ZUFSP L240A,Q241A rev	GTGATCTACAATTGGCTCACCAGGCTGCGCAAGAAGAAGACAGAAAGAGGA

Supplementary Table1: Primers used in this study

Supplementary References:

[1] Alva, V., Nam, S. Z., Söding, J. & Lupas, A. N. The MPI bioinformatics Toolkit as an integrative platform for advanced protein sequence and structure analysis. Nucleic acids research 44, W410-415, (2016)
